# Publisher Correction: *Leishmania* RNA virus exacerbates Leishmaniasis by subverting innate immunity via TLR3-mediated NLRP3 inflammasome inhibition

**DOI:** 10.1038/s41467-019-13900-0

**Published:** 2020-01-07

**Authors:** Renan V. H. de Carvalho, Djalma S. Lima-Junior, Marcus Vinícius G. da Silva, Marisa Dilucca, Tamara S. Rodrigues, Catarina V. Horta, Alexandre L. N. Silva, Patrick F. da Silva, Fabiani G. Frantz, Lucas B. Lorenzon, Marcos Michel Souza, Fausto Almeida, Lilian M. Cantanhêde, Ricardo de Godoi M. Ferreira, Angela K. Cruz, Dario S. Zamboni

**Affiliations:** 1 Departamento de Biologia Celular e Molecular e Bioagentes Patogênicos, Faculdade de Medicina de Ribeirão Preto, Universidade de São Paulo, Ribeirão Preto, São Paulo Brazil; 2 Laboratório de Imunologia e Epigenética, Departamento de Análises Clínicas, Toxicológicas e Bromatologia, Faculdade de Ciências Farmacêuticas de Ribeirão Preto, Universidade de São Paulo, Ribeirão Preto, SP Brazil; 3 Departamento de Bioquímica e Imunologia, Faculdade de Medicina de Ribeirão Preto, Universidade de São Paulo, Ribeirão Preto, São Paulo Brazil; 4 Fundação Oswaldo Cruz, Unidade Rondônia, Porto Velho, Rondônia Brazil

**Keywords:** Parasitic infection, Inflammasome, Monocytes and macrophages, Parasitology, Pathogens

Correction to: *Nature Communications* 10.1038/s41467-019-13356-2, published online 21 November 2019.

The original version of this Article contained errors in Fig. 3, panel L. The second and third lanes should read “L.g.+” and “L.g.−”, respectively. These errors have now been corrected in both the PDF and HTML versions of the Article.

