# Author Correction: *Leishmania* RNA virus exacerbates Leishmaniasis by subverting innate immunity via TLR3-mediated NLRP3 inflammasome inhibition

**DOI:** 10.1038/s41467-025-67433-w

**Published:** 2026-01-05

**Authors:** Renan V. H. de Carvalho, Djalma S. Lima-Junior, Marcus Vinícius G. da Silva, Marisa Dilucca, Tamara S. Rodrigues, Catarina V. Horta, Alexandre L. N. Silva, Patrick F. da Silva, Fabiani G. Frantz, Lucas B. Lorenzon, Marcos Michel Souza, Fausto Almeida, Lilian M. Cantanhêde, Ricardo de Godoi M. Ferreira, Angela K. Cruz, Dario S. Zamboni

**Affiliations:** 1 Departamento de Biologia Celular e Molecular e Bioagentes Patogênicos, Faculdade de Medicina de Ribeirão Preto, Universidade de São Paulo, Ribeirão Preto, São Paulo Brazil; 2 Laboratório de Imunologia e Epigenética, Departamento de Análises Clínicas, Toxicológicas e Bromatologia, Faculdade de Ciências Farmacêuticas de Ribeirão Preto, Universidade de São Paulo, Ribeirão Preto, SP Brazil; 3 Departamento de Bioquímica e Imunologia, Faculdade de Medicina de Ribeirão Preto, Universidade de São Paulo, Ribeirão Preto, São Paulo Brazil; 4 Fundação Oswaldo Cruz, Unidade Rondônia, Porto Velho, Rondônia Brazil

Correction to: *Nature Communications* 10.1038/s41467-019-13356-2, published online 21 November 2019

In the version of the article initially published, there were duplication errors in Fig. 2i, where plots for *L.g*.– 48 h and 96 h were duplicated and where *L.g.+* plots for 24 h and 72 h were duplicated, while in Fig. 6g, an incorrect B-actin plot was displayed. There were also errors in the Source data for Figs. 4h, 6h, 7d and Supplementary Fig. 3c. Corrected versions of Figs. 2 and 6, the Source Data and an explanation of the changes are available in the Supplementary Information accompanying this amendment. Due to the age of the article, the article cannot be updated directly; this amendment serves to correct the article.

## Supplementary information

Revised Figs. 2, 6

Explanation of updates

Revised Source Data

