# Supplementary material for: Leishmania RNA virus exacerbates Leishmaniasis by subverting innate immunity via TLR3-mediated NLRP3 inflammasome inhibition
Source: Nat Commun. 2019 Nov 21;10:5273. doi: 10.1038/s41467-019-13356-2 (PMC6872735; doi:10.1038/s41467-019-13356-2)
Supplement: Supplementary file 1 — Supplementary Information [file 41467_2019_13356_MOESM1_ESM.pdf]

Leishmania RNA virus exacerbates Leishmaniasis by subverting innate immunity via TLR3-mediated NLRP3 inflammasome inhibition

de Carvalho *et al*.

Supplementary Material (Figures and Tables)

Supplem. Figure 1 de Carvalho *et al.*

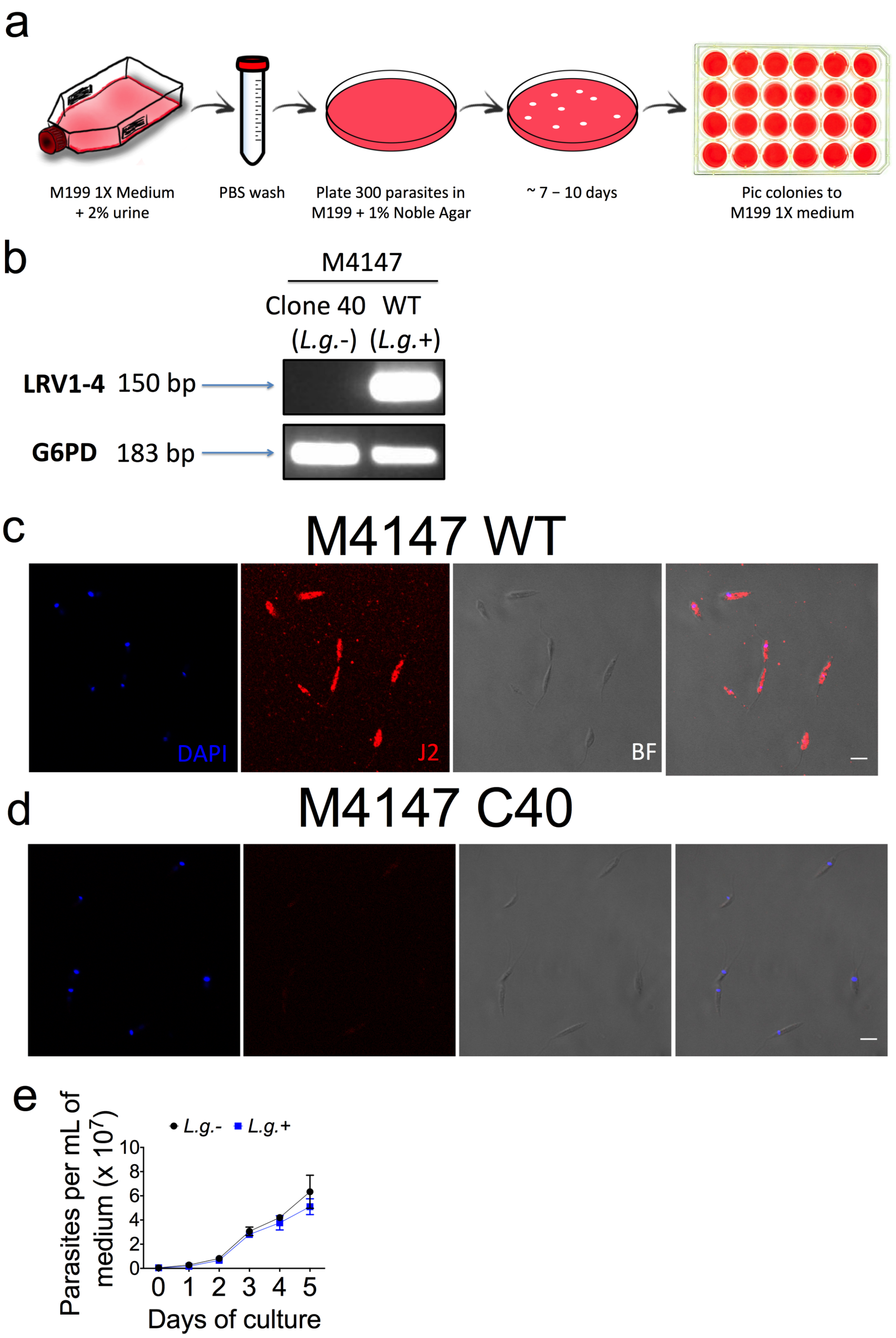

Supplementary Figure 1. Generation of the clone 40 (*L.g.-*). (**a**) Schematics of clone 40 generation (*L.g.-*). (**b**) PCR for LRV product (150 bp) and *Leishmania* G6PD (183 bp) in both clones (*L.g.-* and *L.g.*+). (**c,d**) Confocal microscopy in 8×10^6 fixed and permeabilized promastigotes from the WT strain and its LRV

negative clone 40. Parasites were stained for nuclei (DAPI) and dsRNA (J2 antibody). Scale bar: 5µM. BF: Bright field. (**e**) The growth of both clones was followed in Schneider's insect medium and parasite concentration was determined daily by manual counting in Neubauer's chamber. The results are shown as mean ± SD.

Supplem. Figure 2 de Carvalho *et al.*

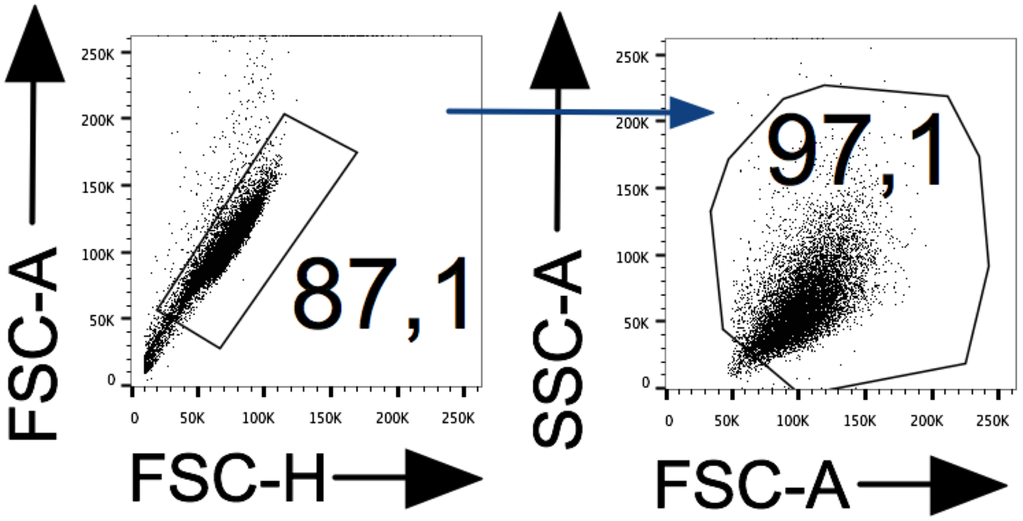

Supplementary Figure 2. Gating strategy in flow-cytometric analysis. Schematics of gating strategy demonstrating how true BMDMs were gated for subsequent analysis of infected cells, cell death and caspase-1 staining. BMDMs were initially gated to exclude debris and doublets (FSC-A/FSC-H). Then, true BMDMs were gated according to their granularity and size (SSC-A/FSC-A). Next, cells were gated according to the objective/staining in each experiment of the current study.

Supplem. Figure 3 de Carvalho *et al.*

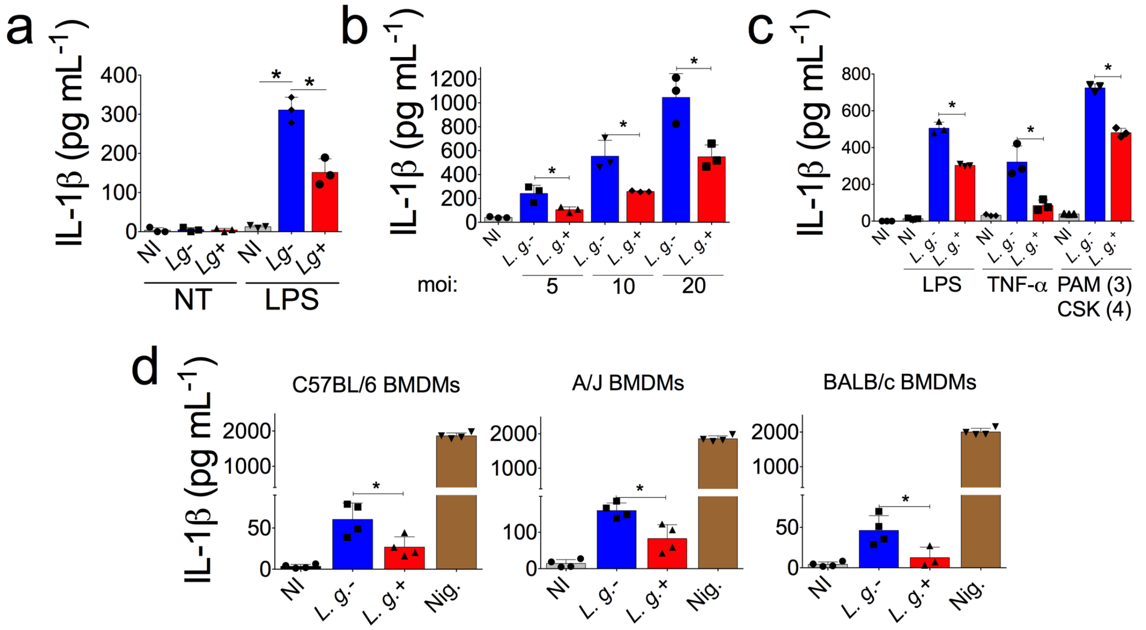

Supplementary Figure 3. LRV attenuates inflammasome activation by *L.g.*
(**a**) C57BL/6 BMDMs were left unprimed or primed with LPS (500 ng/mL) for 4 hours, and then infected with SP Promastigotes of *L.g.* clones at MOI 10. (**b**) LPS-primed (500 ng/mL) BMDMs were infected with three different MOI of SP Promastigotes of *L.g.-* or *L.g.+*. (**c**) Cells were primed for 4h with either LPS (500 ng/mL), PAM(3)CSK(4) (300 ng/mL) or TNF-α (10 ng/mL), and then infected for 24h with *L.g.- or L.g.+*. (**d**) BMDMs from different mouse strains were primed with LPS and infected for 24h with *L.g.* clones, or treated with 20 μM of Nigericin (positive control). After 24 hours, supernatants were collected and ELISA for IL-1β was performed in cell-free supernatants. One representative of at least two independent experiments performed with technical replicates is shown. The results are shown as mean ± SD. Statistical analysis was performed by unpaired Student's *t test*, and $P<0.05$ (*) was considered statistically significant.

Supplem. Figure 4 de Carvalho *et al.*

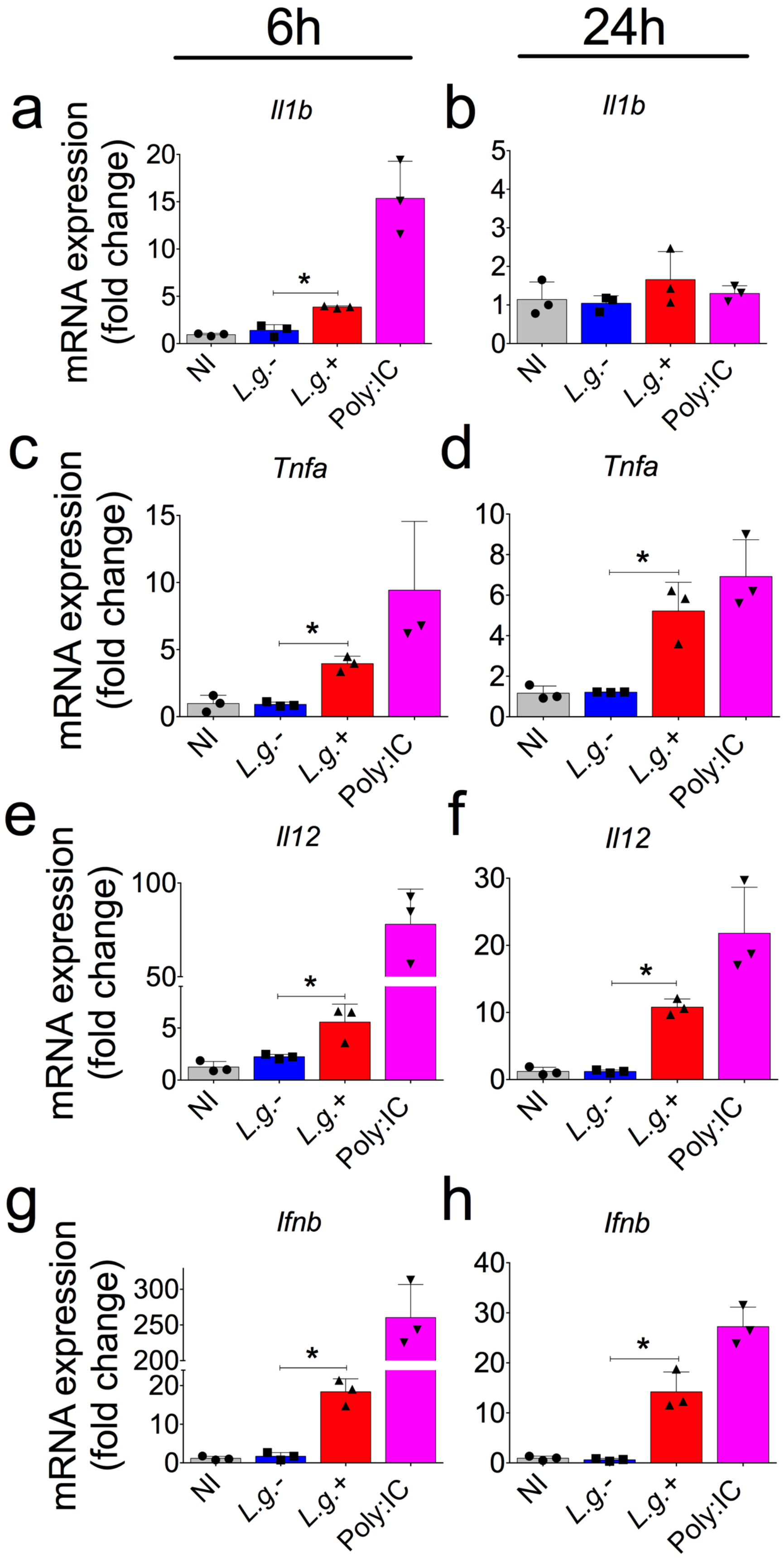

Supplementary Figure 4. LRV induces transcription of different inflammatory genes. C57BL/6 BMDMs were infected with *L.g.*- or *L.g.*+ for 6 (**a,c,e,g**) or 24 hours (**b,d,f,h**). qPCR for *Il1b* (**a,b**), *Tnfa* (**c,d**), *Il12* (**e,f**) and *Ifnb* (**g,h**) was performed. Poly:IC (5 μg/mL) was used as a positive control. One representative of two independent experiments performed with technical replicates is shown. The results are shown as mean ± SD. Statistical analysis was performed by unpaired Student's *t test*, and $P<0.05$ (*) was considered statistically significant.

Supplem. Figure 5 de Carvalho *et al.*

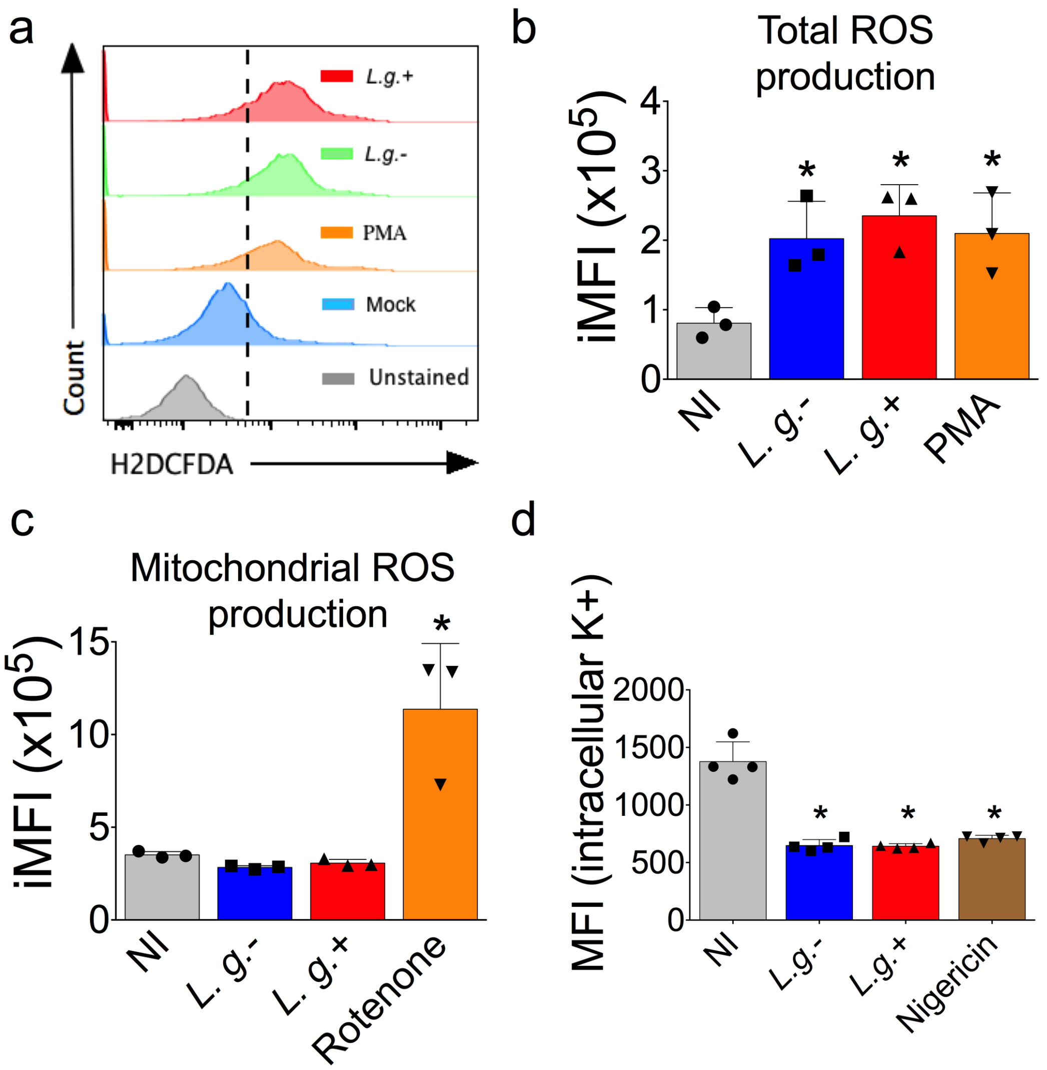

Supplementary Figure 5. LRV does not interfere with the generation of second signals by *L.g.* (**a-c**) C57BL/6 BMDMs were infected with either *L.g.-* or *L.g.+* for 90 minutes. Then, a fluorescent dye staining total (**a,b**) or mitochondrial (**c**) ROS was added for additional 30 minutes. Cells were harvested and analyzed by FACS. (**d**) Cells were incubated with APG-2 dye and 12 hours after infection, levels of intracellular potassium were determined by a High Content Screening Confocal equipment. The results are shown as mean ± SD. Statistical analysis was performed by unpaired Student's *t test*, and *P*<0.05 (*) was considered statistically significant. One representative of two independent experiments performed with technical replicates is shown.

Supplem. Figure 6 de Carvalho *et al.*

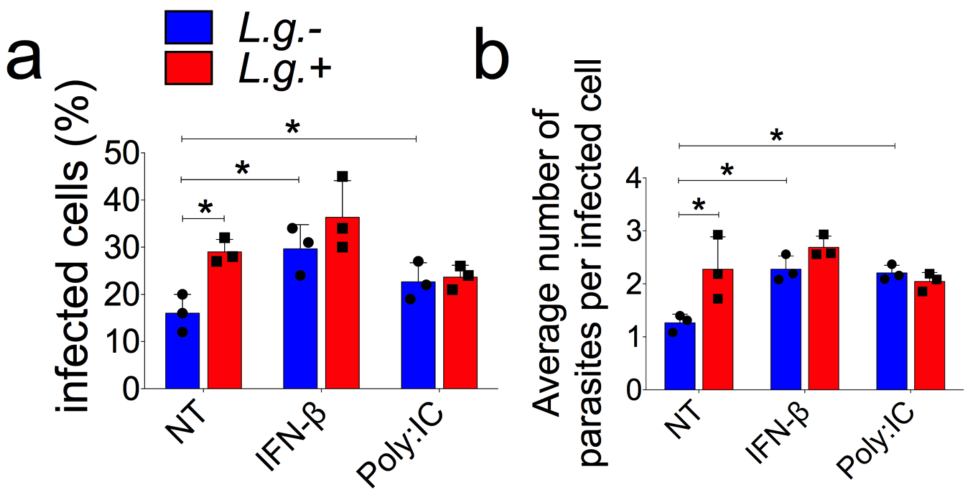

Supplementary Figure 6. TLR3, TRIF, IFN-β and NLRP3 are required for LRV-mediated parasite survival. (**a,b**) C57BL/6 BMDMs were infected with metacyclic parasites from either *L.g.-* or *L.g.+* at a MOI of 1. After 1h of infection, cells were washed and replaced with fresh medium containing either Poly:IC (5 μg/mL) (I) or IFN-β (1000 U/mL), and left for 48 hours in culture. The results are shown as mean ± SD. Statistical analysis was performed by unpaired Student's *t test*, and *P*<0.05 (*) was considered statistically significant. One representative of two independent experiments performed with technical replicates is shown.

Supplem. Figure 7 de Carvalho *et al.*

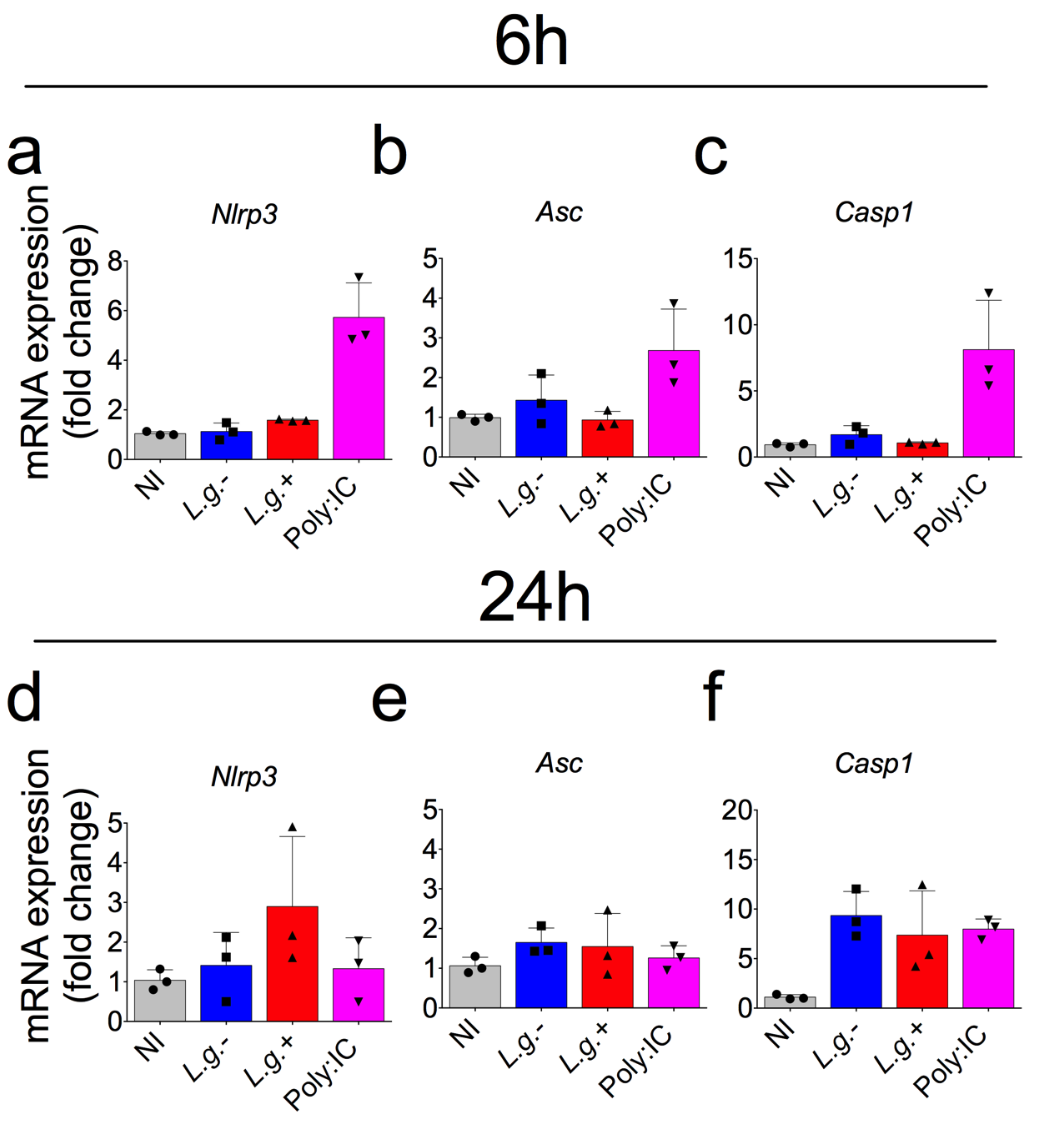

Supplementary Figure 7. LRV does not alter the transcription of inflammasome components. C57BL/6 BMDMs were infected with *L.g.*- or *L.g.*+. After 6 (**a-c**) or 24h of infection (**d-f**), total RNA was extracted. Levels of *Nlrp3* (**a,d**)*, Asc* (**b,e**) and *casp1* (**c,f**) transcripts were determined by qPCR analysis. Poly:IC (5 μg/mL) was used as a positive control. The results are shown as mean ± SD. One representative of two independent experiments performed with technical replicates is shown.

Supplem. Figure S8 de Carvalho *et al.*

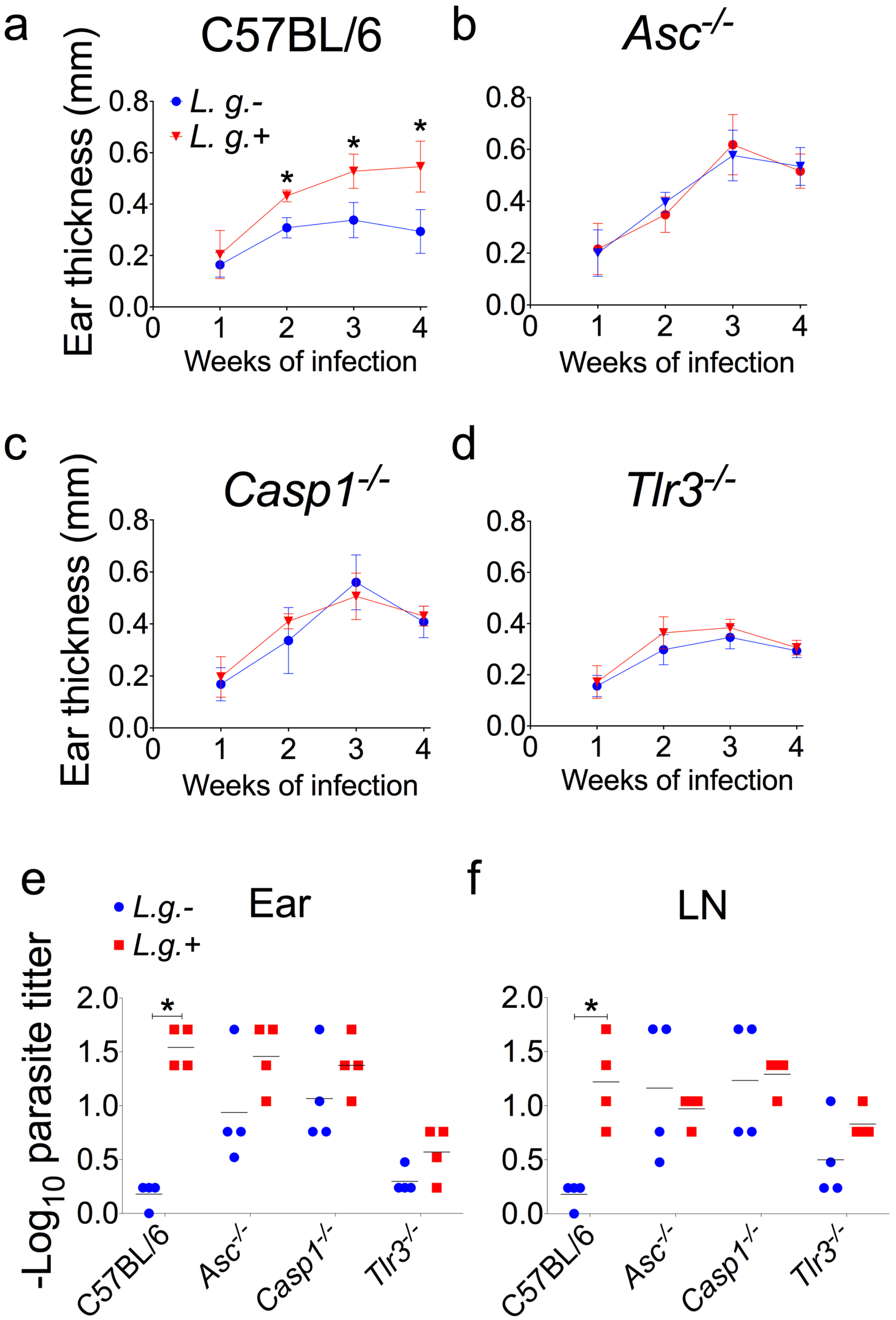

Supplementary Figure 8. LRV exacerbates *L.g.* infection via ASC, CASP1 and TLR3 *in vivo*. (**a-d**) C57BL/6 (**a**), *Asc*$^{-/-}$ (**b**), *Casp1*$^{-/-}$ (**c**) and *Tlr3*$^{-/-}$ (**d**) mice were injected with 10^6 Stationary-phase promastigotes of *L.g.*- or *L.g.*+ (n = 4-5

mice per group), and ear thicknesses were followed weekly (**a-d**). 4 weeks after infection, parasite titers were determined in the ear (**e**) and draining lymph node (**f**) from all mice used in this experiment. The results are shown as mean **(e,f)** or mean ± SD **(a,b,c,d)** from data obtained from two independent experiments performed with biological replicates. Statistical analysis was performed by two-way ANOVA with Bonferroni's multiple comparison test. *P*<0.05 (*) was considered statistically significant.

Supplem. Table 1 de Carvalho *et al.*

Patient number	Infectious specie	Region	Outcome	LRV Status
1	*L. braziliensis*	Rondônia, Brazil	CL	POSITIVE
2	*Leishmania spp.*	Rondônia, Brazil	CL	NEGATIVE
3	*Leishmania spp.*	Rondônia, Brazil	MCL	NEGATIVE
4	*Leishmania spp.*	Rondônia, Brazil	CL	NEGATIVE
5	*L. braziliensis*	Rondônia, Brazil	CL	POSITIVE
6	*L. braziliensis*	Rondônia, Brazil	CL	NEGATIVE
7	*L. braziliensis*	Rondônia, Brazil	CL	POSITIVE
8	*L. braziliensis*	Rondônia, Brazil	CL	POSITIVE
9	*Leishmania spp.*	Rondônia, Brazil	CL	NEGATIVE
10	*Leishmania spp.*	Rondônia, Brazil	CL	NEGATIVE
11	*Leishmania spp.*	Rondônia, Brazil	CL	NEGATIVE
12	*L. braziliensis*	Rondônia, Brazil	MCL	POSITIVE
13	*L. braziliensis*	Rondônia, Brazil	CL	POSITIVE
14	*L. braziliensis*	Rondônia, Brazil	CL	NEGATIVE
15	*Leishmania spp.*	Rondônia, Brazil	CL	NEGATIVE
16	*Leishmania spp.*	Rondônia, Brazil	CL	NEGATIVE
17	*Leishmania spp.*	Rondônia, Brazil	CL	POSITIVE
18	*L. guyanensis*	Rondônia, Brazil	CL	NEGATIVE
19	*L. braziliensis*	Rondônia, Brazil	MCL	POSITIVE
20	*L. braziliensis*	Rondônia, Brazil	CL	POSITIVE
21	*L. guyanensis*	Rondônia, Brazil	CL	POSITIVE
22	*L. braziliensis*	Rondônia, Brazil	CL	NEGATIVE
23	*L. braziliensis*	Rondônia, Brazil	CL	NEGATIVE
24	*Leishmania spp.*	Rondônia, Brazil	CL	NEGATIVE
25	*L. braziliensis*	Rondônia, Brazil	CL	NEGATIVE
26	*L. braziliensis*	Rondônia, Brazil	MCL	NEGATIVE
27	*Leishmania spp.*	Rondônia, Brazil	CL	NEGATIVE
28	*L. braziliensis*	Rondônia, Brazil	MCL	POSITIVE
29	*Leishmania spp.*	Rondônia, Brazil	MCL	POSITIVE
30	*Leishmania spp.*	Rondônia, Brazil	CL	NEGATIVE
31	*Leishmania spp.*	Rondônia, Brazil	CL	NEGATIVE
32	*L. braziliensis*	Rondônia, Brazil	CL	POSITIVE
33	*L. guyanensis*	Rondônia, Brazil	MCL	NEGATIVE
34	*Leishmania spp.*	Rondônia, Brazil	CL	NEGATIVE
35	*L. braziliensis*	Rondônia, Brazil	MCL	POSITIVE
36	*L. braziliensis*	Rondônia, Brazil	MCL	POSITIVE
37	*L. braziliensis*	Rondônia, Brazil	CL	NEGATIVE
38	*Leishmania spp.*	Rondônia, Brazil	CL	NEGATIVE
39	*L. braziliensis*	Rondônia, Brazil	CL	POSITIVE
40	*L. braziliensis*	Rondônia, Brazil	CL	POSITIVE
41	*Leishmania spp.*	Rondônia, Brazil	CL	NEGATIVE
42	*L. guyanensis*	Rondônia, Brazil	CL	NEGATIVE
43	*L. braziliensis*	Rondônia, Brazil	CL	POSITIVE

Supplementary Table 1. Cervical brushes obtained from patients. The diagram shows the distribution of the samples tested in figure 1, according to the specie of the infecting parasite, place of Isolation, outcome of the disease (CL= Cutaneous Leishmaniasis; MCL = Mucocutaneous Leishmaniasis), and LRV status.

Supplem. Table 2 de Carvalho *et al.*

Collection Label	Specie	Place of Isolation	LRV Status
IOCL 3569 (299)	*L. braziliensis*	Rondônia, Brazil	POSITIVE
IOCL 3570 (303)	*L. braziliensis*	Rondônia, Brazil	POSITIVE
IOCL 3571 (304)	*L. braziliensis*	Rondônia, Brazil	POSITIVE
IOCL 3622 (476)	*L. braziliensis*	Rondônia, Brazil	POSITIVE
IOCL 3625 (313)	*L. braziliensis*	Rondônia, Brazil	POSITIVE
IOCL 3639 (390)	*L. braziliensis*	Rondônia, Brazil	POSITIVE
IOCL 3626 (314)	*L. braziliensis*	Rondônia, Brazil	NEGATIVE
IOCL 3642 (412)	*L. braziliensis*	Rondônia, Brazil	NEGATIVE
IOCL 3710 (666)	*L. braziliensis*	Rondônia, Brazil	POSITIVE
IOCL 3708 (667)	*L. braziliensis*	Rondônia, Brazil	NEGATIVE
IOCL 3714 (760)	*L. braziliensis*	Rondônia, Brazil	NEGATIVE
IOCL 3713 (767)	*L. braziliensis*	Rondônia, Brazil	NEGATIVE
IOCL 3711 (818)	*L. braziliensis*	Rondônia, Brazil	POSITIVE
IOCL 3712 (820)	*L. braziliensis*	Rondônia, Brazil	NEGATIVE
IOCL 3637 (384)	*L. braziliensis*	Rondônia, Brazil	POSITIVE
IOCL 3540 (268)	*L. braziliensis*	Rondônia, Brazil	POSITIVE
IOCL 3539 (271)	*L. guyanensis*	Rondônia, Brazil	POSITIVE
IOCL 3460 (391)	*L. guyanensis*	Rondônia, Brazil	NEGATIVE
IOCL 3546 (276)	*L. braziliensis*	Rondônia, Brazil	NEGATIVE
IOCL 3538 (251)	*L. guyanensis*	Rondônia, Brazil	NEGATIVE
IOCL 3549 (283)	*L. braziliensis*	Rondônia, Brazil	NEGATIVE
IOCL 3567 (291)	*L. braziliensis*	Rondônia, Brazil	POSITIVE
IOCL 3545 (275)	*L. braziliensis*	Rondônia, Brazil	POSITIVE

Supplementary Table 2. Clinical isolates obtained from patients. The diagram shows the distribution of the *L.b.* and *L.g.* parasites tested in figure 10, according to their International code, FIOCRUZ collection label and LRV status.
