# Supplementary figures and images for: Author Correction: Leishmania RNA virus exacerbates Leishmaniasis by subverting innate immunity via TLR3-mediated NLRP3 inflammasome inhibition
Source: Nat Commun. 2026 Jan 5;17:105. doi: 10.1038/s41467-025-67433-w (PMC12770502; doi:10.1038/s41467-025-67433-w)

Figure 2

## SP Promastigotes

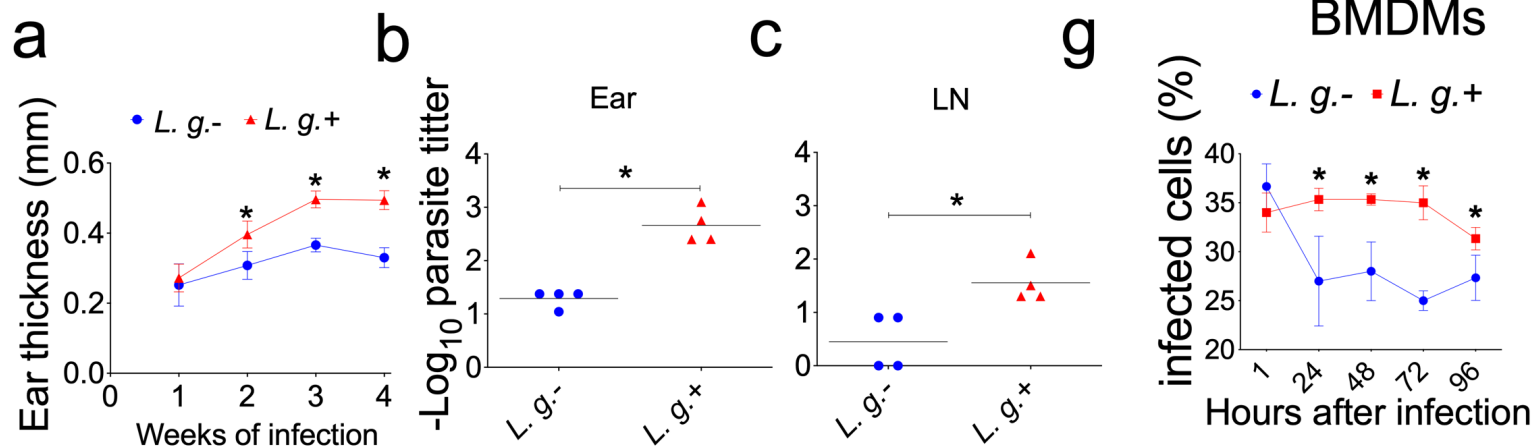

## Metac. Promastigotes

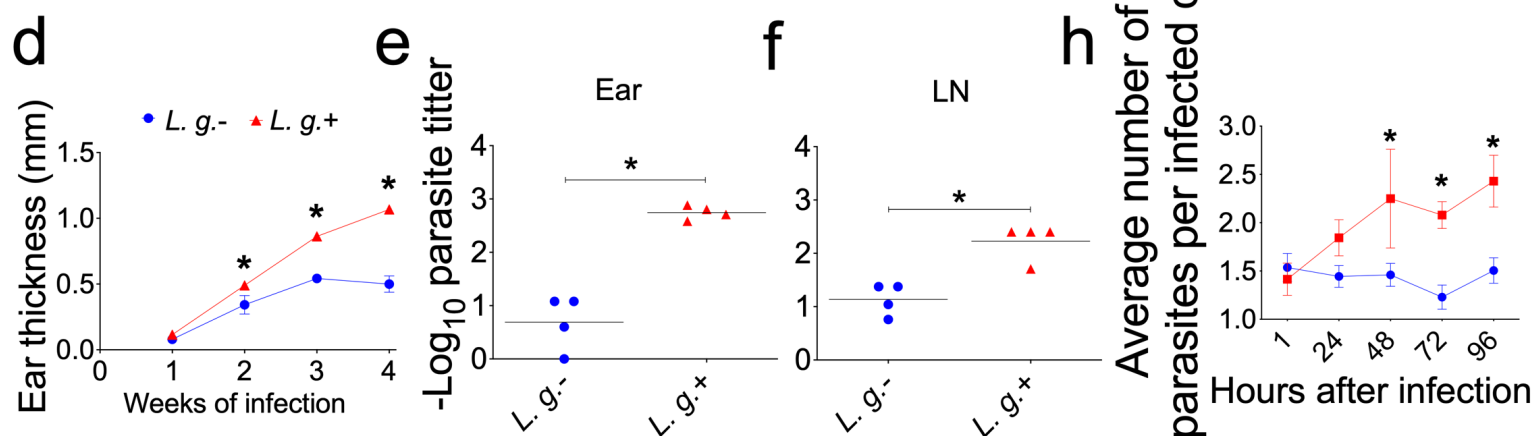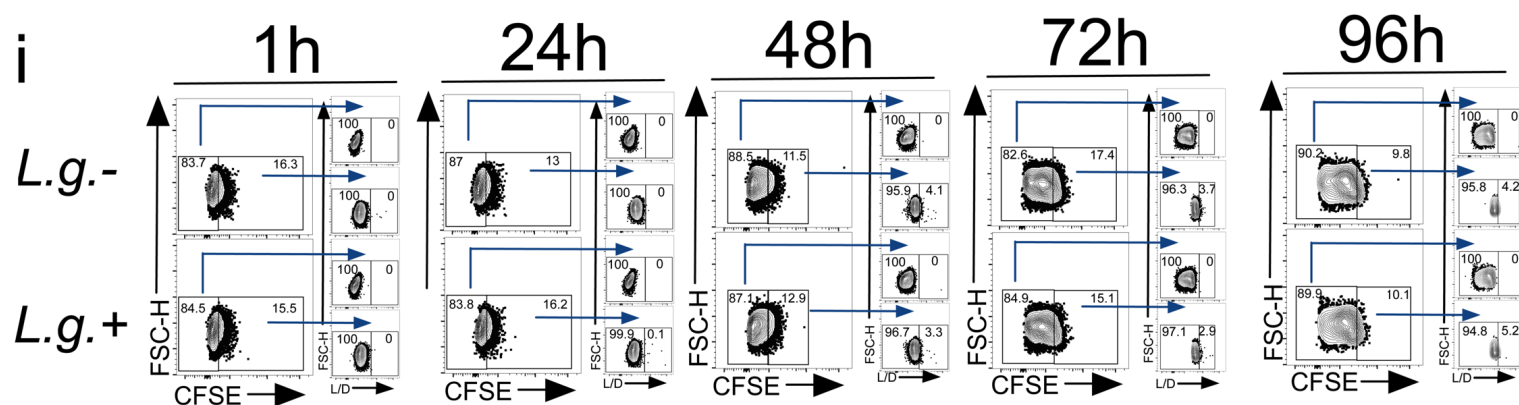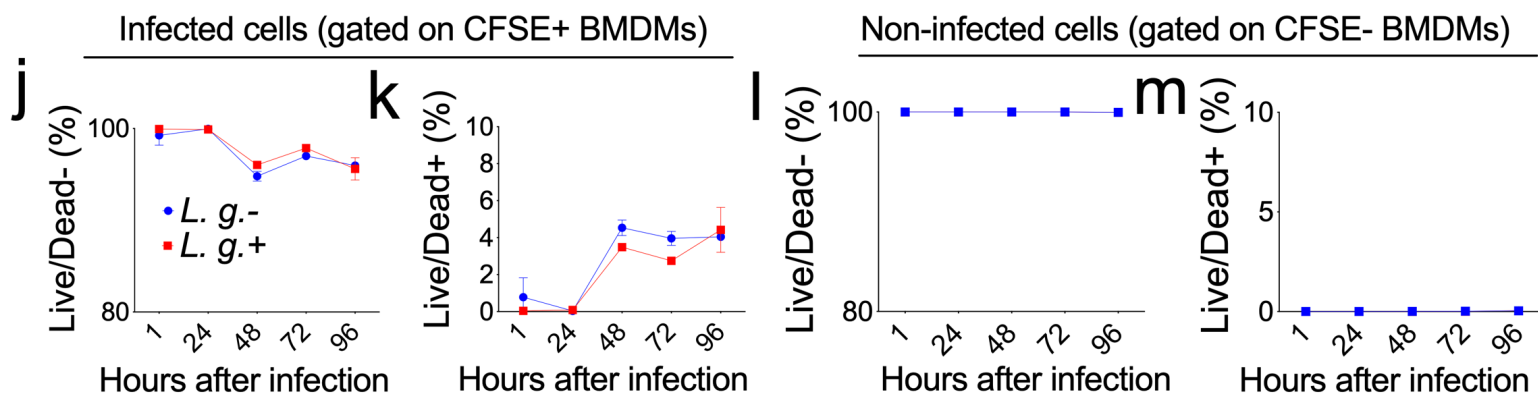

Figure 6

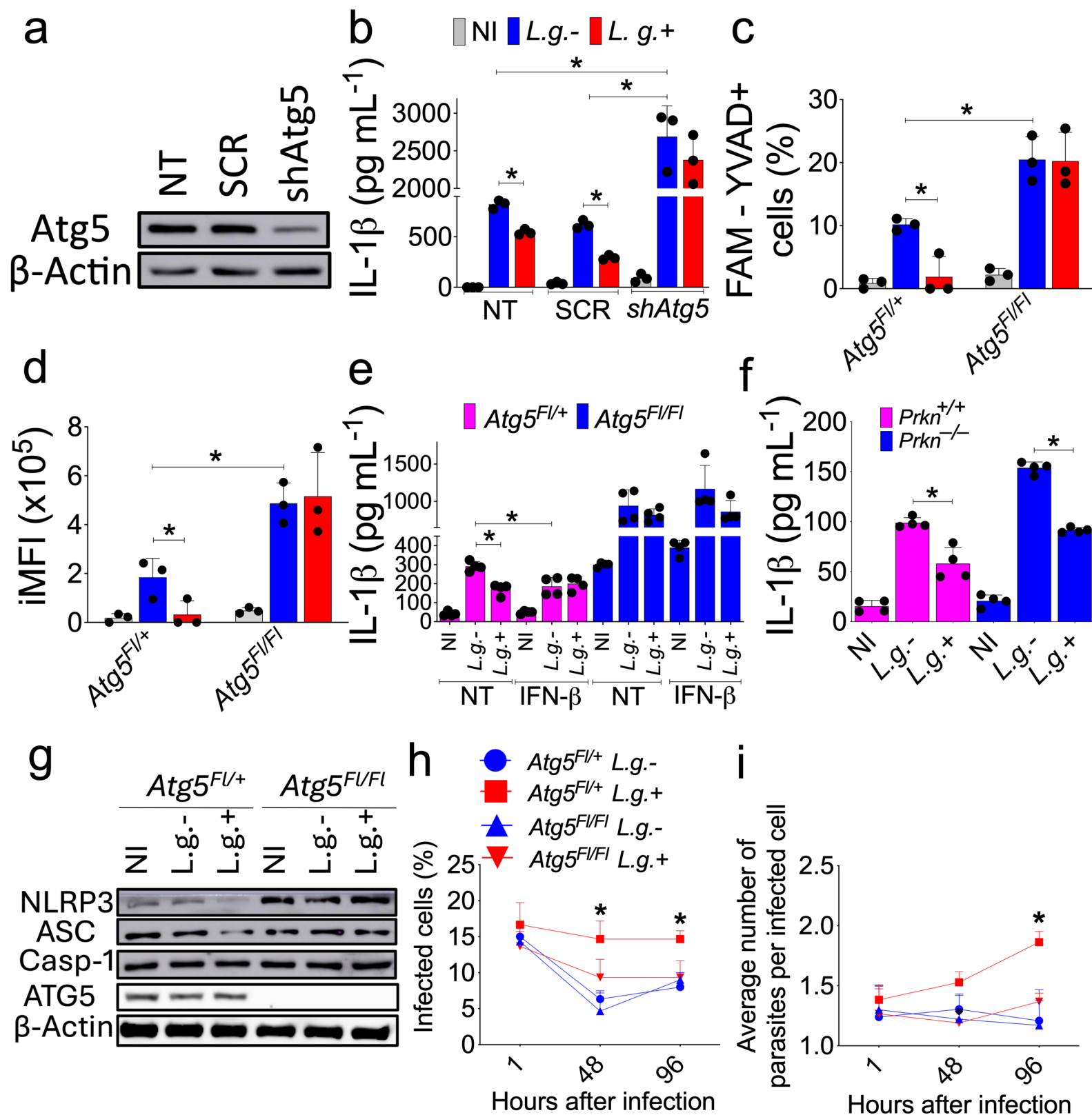

Supplement: Supplementary file 1 — Revised Figs. 2, 6 [file 41467_2025_67433_MOESM1_ESM.pdf]
