# Supplementary material for: Author Correction: Leishmania RNA virus exacerbates Leishmaniasis by subverting innate immunity via TLR3-mediated NLRP3 inflammasome inhibition
Source: Nat Commun. 2026 Jan 5;17:105. doi: 10.1038/s41467-025-67433-w (PMC12770502; doi:10.1038/s41467-025-67433-w)
Supplement: Supplementary file 2 — Explanation of updates [file 41467_2025_67433_MOESM2_ESM.pdf]

❌ Current figure 2i:
Wrong final figure,
blots were duplicated

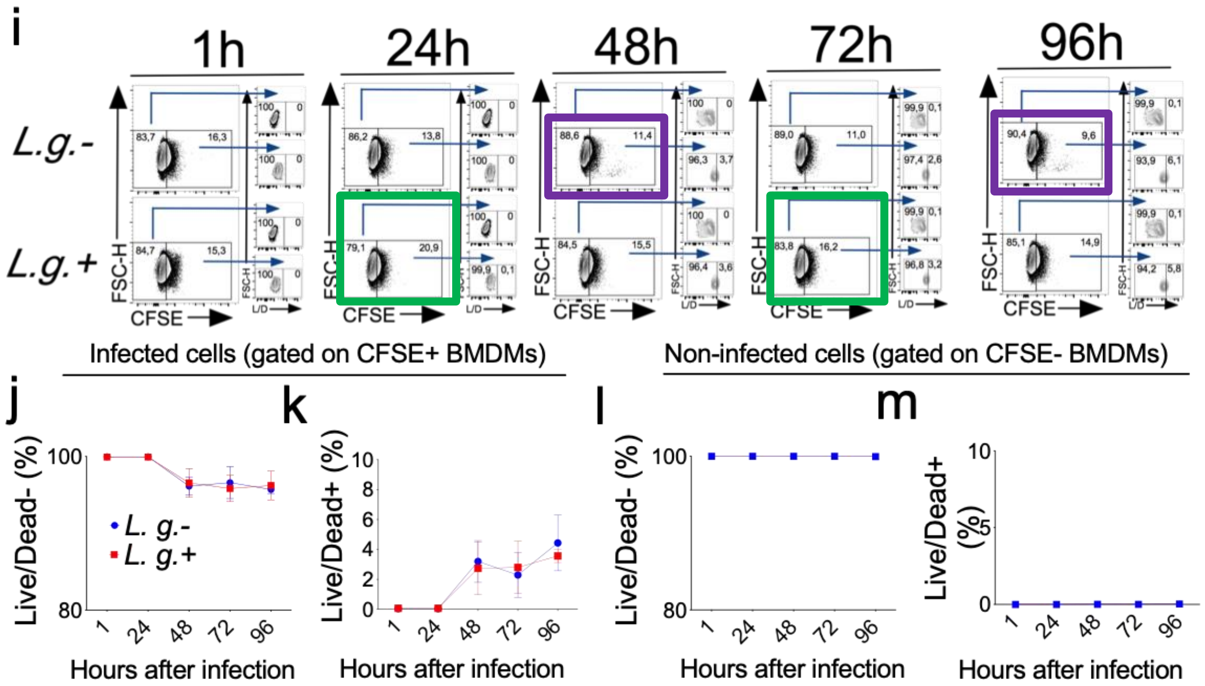

✅ New figure 2:
Original exp reanalyzed,
yielding similar results

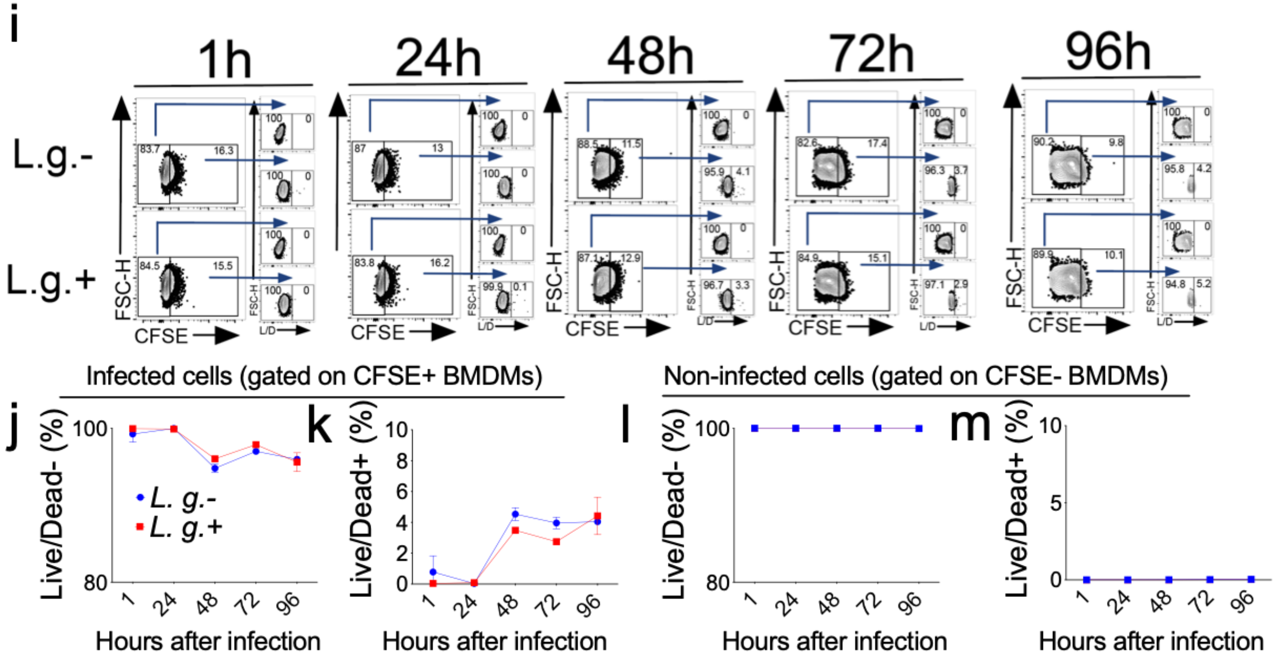

Current figure 6g:
Actin blot is not the original

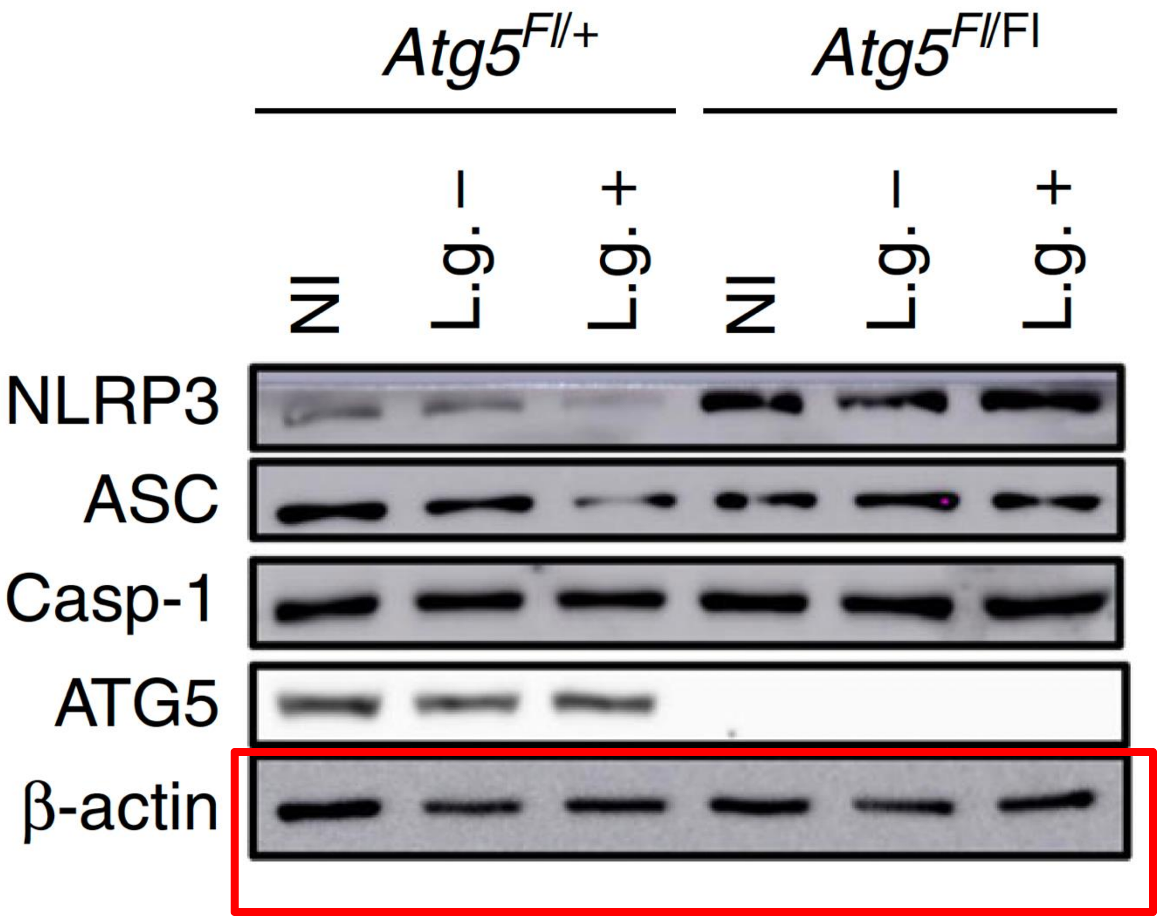

New figure 6g, with the original Actin blot

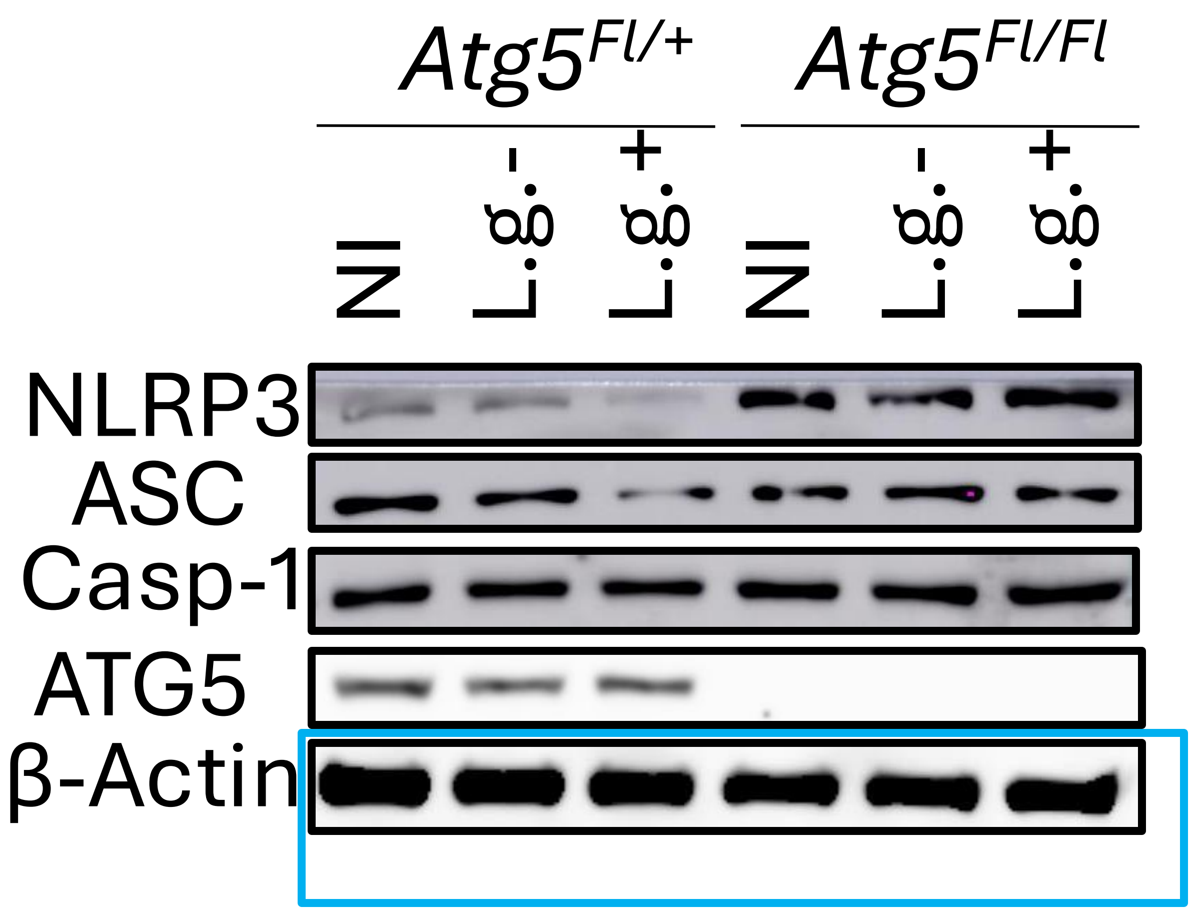

❌ Current source data file:
Label in Fig 4h wrongly annotated as Poly IC

Fig 4g	NT	NT	NT	Poly:IC	Poly:IC	Poly:IC
	NI	L.g.-	L.g.+	NI	L.g.-	L.g.+
	21.45	764.36	391.96	0.00	284.00	402.36
	0.00	716.69	473.14	14.26	309.90	361.53
	0.00	698.92	422.02	17.53	369.30	363.88

Fig 4h	NT	NT	NT	Poly:IC	Poly:IC	Poly:IC
	NI	L.g.-	L.g.+	NI	L.g.-	L.g.+
	13.00	121.00	99.00	6.00	84.00	96.00
	5.00	141.00	73.00	8.00	63.00	62.00
	22.00	135.00	79.00	22.00	62.00	137.00

✅ New source data file:
Labeling now corresponds to Fig. 4h (IFN-b)

Fig 4g	NT	NT	NT	Poly:IC	Poly:IC	Poly:IC
	NI	L.g.-	L.g.+	NI	L.g.-	L.g.+
	21.45	764.36	391.96	0.00	284.00	402.36
	0.00	716.69	473.14	14.26	309.90	361.53
	0.00	698.92	422.02	17.53	369.30	363.88

Fig 4h	NT	NT	NT	IFN-b	IFN-b	IFN-b
	NI	L.g.-	L.g.+	NI	L.g.-	L.g.+
	13.00	121.00	99.00	6.00	84.00	96.00
	5.00	141.00	73.00	8.00	63.00	62.00
	22.00	135.00	79.00	22.00	62.00	137.00

Current source data file:
Numbers shown are not from experiment presented in Fig. 6h

Fig 6h		Atg5+/- L. g.-			Atg5+/- L. g.+			Atg5-/- L. g.-			Atg5-/- L. g.+		
	1	55.00	58.00	50.00	56.00	54.00	58.00	50.00	54.00	55.00	53.00	56.00	51.00
	48	30.00	34.00	27.00	49.00	43.00	63.00	26.00	32.00	30.00	34.00	43.00	38.00
	96	19.00	17.00	15.00	40.00	39.00	49.00	22.00	16.00	16.00	26.00	26.00	27.00

New source data file:
Numbers shown are now corrected and show data from Fig. 6h

Fig 6h		Atg5+/- L. g.-			Atg5+/- L. g.+			Atg5-/- L. g.-			Atg5-/- L. g.+		
	1	13	15	17	14	16	20	14	12	17	16	12	13
	48	7	7	5	12	15	17	2	5	7	12	7	9
	96	10	6	8	14	14	16	10	8	9	12	8	8

__Current source data file:__
Numbers from Figure 7c were duplicated in Fig. 7d when copy-pasting results

Fig 7c	C57BL/6	NT	NT	NT	EVs Lg+	EVs Lg+	EVs Lg+	EVs Lg-	EVs Lg-	EVs Lg-
		NI	*L.g.-*	*L.g.+*	NI	*L.g.-*	*L.g.+*	NI	*L.g.-*	*L.g.+*
		0.00	244.25	127.88	0.00	111.85	144.25	1.24	230.04	122.74
		0.00	238.02	127.07	0.00	138.16	222.47	0.00	336.53	122.74
		0.00	242.35	95.31	2.46	148.86	117.34	0.00	242.76	223.69
	Tlr3-/-	NT	NT	NT	EVs Lg+	EVs Lg+	EVs Lg+	EVs Lg-	EVs Lg-	EVs Lg-
		NI	*L.g.-*	*L.g.+*	NI	*L.g.-*	*L.g.+*	NI	*L.g.-*	*L.g.+*
		0.00	180.48	165.86	8.14	174.60	141.54	4.15	154.68	241.98
		0.00	184.05	164.50	6.23	169.13	145.74	7.35	234.15	133.15
		0.00	173.10	159.05	14.37	175.97	157.96	22.57	201.35	194.20
Fig 7d	Atg5Fl/+	NT	NT	NT	EVs Lg+	EVs Lg+	EVs Lg+	EVs Lg-	EVs Lg-	EVs Lg-
		NI	*L.g.-*	*L.g.+*	NI	*L.g.-*	*L.g.+*	NI	*L.g.-*	*L.g.+*
		0.00	244.25	127.88	0.00	111.85	144.25	1.24	230.04	122.74
		0.00	238.02	127.07	0.00	138.16	222.47	0.00	336.53	122.74
		0.00	242.35	95.31	2.46	148.86	117.34	0.00	242.76	223.69
	Atg5Fl/Fl	NT	NT	NT	EVs Lg+	EVs Lg+	EVs Lg+	EVs Lg-	EVs Lg-	EVs Lg-
		NI	*L.g.-*	*L.g.+*	NI	*L.g.-*	*L.g.+*	NI	*L.g.-*	*L.g.+*
		0.00	180.48	165.86	8.14	174.60	141.54	4.15	154.68	241.98
		0.00	184.05	164.50	6.23	169.13	145.74	7.35	234.15	133.15
		0.00	173.10	159.05	14.37	175.97	157.96	22.57	201.35	194.20

__New source data file:__
Raw data from Figure 7d correspond to what is seen in the original graph

Fig 7c	C57BL/6	NT	NT	NT	EVs Lg+	EVs Lg+	EVs Lg+	EVs Lg-	EVs Lg-	EVs Lg-
		NI	*L.g.-*	*L.g.+*	NI	*L.g.-*	*L.g.+*	NI	*L.g.-*	*L.g.+*
		0.00	244.25	127.88	0.00	111.85	144.25	1.24	230.04	122.74
		0.00	238.02	127.07	0.00	138.16	222.47	0.00	336.53	122.74
		0.00	242.35	95.31	2.46	148.86	117.34	0.00	242.76	223.69
	Tlr3-/-	NT	NT	NT	EVs Lg+	EVs Lg+	EVs Lg+	EVs Lg-	EVs Lg-	EVs Lg-
		NI	*L.g.-*	*L.g.+*	NI	*L.g.-*	*L.g.+*	NI	*L.g.-*	*L.g.+*
		0.00	180.48	165.86	8.14	174.60	141.54	4.15	154.68	241.98
		0.00	184.05	164.50	6.23	169.13	145.74	7.35	234.15	133.15
		0.00	173.10	159.05	14.37	175.97	157.96	22.57	201.35	194.20
Fig 7d	Atg5Fl/+	NT	NT	NT	EVs Lg+	EVs Lg+	EVs Lg+	EVs Lg-	EVs Lg-	EVs Lg-
		NI	*L.g.-*	*L.g.+*	NI	*L.g.-*	*L.g.+*	NI	*L.g.-*	*L.g.+*
		17.474	221.704	105.53	21.258	92.029	73.642	20.717	306.88	108.02
		9.491	267.521	94.907	12.335	111.146	65.724	20.853	328.47	118.98
		5.417	226.348	92.016	11.658	143.403	65.042	21.528	329.38	122.87
	Atg5Fl/Fl	NT	NT	NT	EVs Lg+	EVs Lg+	EVs Lg+	EVs Lg-	EVs Lg-	EVs Lg-
		NI	*L.g.-*	*L.g.+*	NI	*L.g.-*	*L.g.+*	NI	*L.g.-*	*L.g.+*
		249.88	983.911	1057.8	251.1767	969.786	966.291	101.47	919.99	863.45
		254.01	751.339	1009.3	201.672	718.94	884.787	461.11	942.06	873.36
		159.13	996.8246	978.96	435.6193	810.884	901.0037	418.85	645.35	858.7

Current Fig. S3c source data file:
Missing NI, Lg- and Lg+ labels under treatments

Fig S3c										
	NP	LPS			TNF-a			PAM(3)		
	0.00	13.15	495.47	301.75	29.46	259.56	118.36	38.93	733.95	477.80
	0.00	17.46	541.99	297.68	37.23	284.30	60.47	38.09	740.38	459.49
	0.00	10.15	479.78	310.02	28.99	421.31	75.63	40.21	700.47	506.56

New source data file for Fig. S3c:
Labels were added

Fig S3c		LPS			TNF-a			PAM(3)		
	NP	NI	L.g.-	L.g.+	NI	L.g.-	L.g.+	NI	L.g.-	L.g.+
	0.00	13.15	495.47	301.75	29.46	259.56	118.36	38.93	733.95	477.80
	0.00	17.46	541.99	297.68	37.23	284.30	60.47	38.09	740.38	459.49
	0.00	10.15	479.78	310.02	28.99	421.31	75.63	40.21	700.47	506.56
